# An Autobiographical Case Series of Familial Post Ambulatory Swollen Hands (POTASH): Hand Swelling in a Man and His Sister While Participating in a Half Marathon

**DOI:** 10.7759/cureus.55518

**Published:** 2024-03-04

**Authors:** Philip R Cohen, Bonnie S Cohen, Razelle Kurzrock

**Affiliations:** 1 Dermatology, University of California, Davis Medical Center, Sacramento, USA; 2 Dermatology, Touro University California College of Osteopathic Medicine, Vallejo, USA; 3 Periodontics, Nova Southeastern University Dental School, Fort Lauderdale, USA; 4 Medicine, Medical College of Wisconsin Cancer Center and Genome Sciences and Precision Medicine Center, Milwaukee, USA; 5 Oncology, Worldwide Innovative Network (WIN) Consortium, Villejuif, FRA

**Keywords:** swelling, sporadic, running, potash, post ambulatory, marathon, hand, finger, familial, ambulatory

## Abstract

Post ambulatory swollen hands (POTASH) is an acquired condition characterized by swelling of the hands, thumbs, and fingers following either walking, hiking, or running; no other body sites are swollen. The asymptomatic hand swelling begins in adulthood and recurs after adequate ambulation. A distinctive feature of POTASH that is often present is a positive fist sign demonstrated by the inability of the affected person to clench their fingers into the palm and form a fist. POTASH usually resolves spontaneously within a few hours after stopping ambulation; however, less frequently, it can persist for one or two days. The pathogenesis of POTASH has not been determined. In this case report, POTASH is described in an adult man and his sister. Neither the man's parents nor two of his other younger sisters had POTASH. However, the man's wife also develops POTASH with prolonged exercise; none of the three biological adult children of the man and his wife had POTASH. Therefore, based on these observations, the possibility that POTASH may have an autosomal recessive mode of inheritance and/or may be sporadic is suggested.

## Introduction

Post ambulatory swollen hands (POTASH) is swelling of the hands elicited by ambulating [1]. It was originally described as big hand syndrome by Brazilian researchers who were evaluating hand swelling in individuals who walked their dogs [2]. Subsequently, it has been described not only in hikers but also in runners and walkers [1,3,4].

POTASH has repeatedly been observed in a half marathon participant. The hand swelling is asymptomatic and typically begins after an hour of either running or walking. In addition, a positive fist sign is usually demonstrated by the fingers of the affected hands not being able to be clenched into a fist [1,4,5].

The pathogenesis of POTASH remains to be established. Patient factors and environmental factors may have an influence on the development of POTASH. Potential environmental factors might include pollens, chemical agents, viruses, and other antigens [1,2,4].

A mode of inheritance of POTASH has not been described [1,2,4]. A man, his sister, and his wife each developed POTASH while walking a half marathon. Neither the man's parents nor his other two sisters nor his biological children have POTASH. The possibility that POTASH may be inherited as an autosomal recessive condition and/or may be a sporadic condition is suggested.

## Case presentation

Case 1

A 65-year-old man walked a 13.1-mile half marathon in January 2023 in the suburban community of Sugar Land, Texas; the race location was neither along a river, nor along the seashore, nor in the mountains. The temperature at the start of the race was 44 degrees Fahrenheit. The ambient temperature had only increased to 48 degrees Fahrenheit.

His hands started to swell after he had been walking for one hour. The asymptomatic swelling continued during the remainder of the race; there was no itching, and no other areas became swollen. He had been a long-distance runner since high school and had run 20 marathons and numerous half marathons; however, his initial episode of recurrent post ambulatory hand swelling occurred about seven years ago, at age 58 years, after participating in a half marathon [1,4,5].

Neither of his parents has POTASH. Also, two of his younger sisters who had previously run one or more marathons or half marathons or both, currently ages 63 years and 61 years, did not have POTASH. However, his youngest sister (Case 2), who is 58 years of age, also has POTASH.

**Figure 1:**
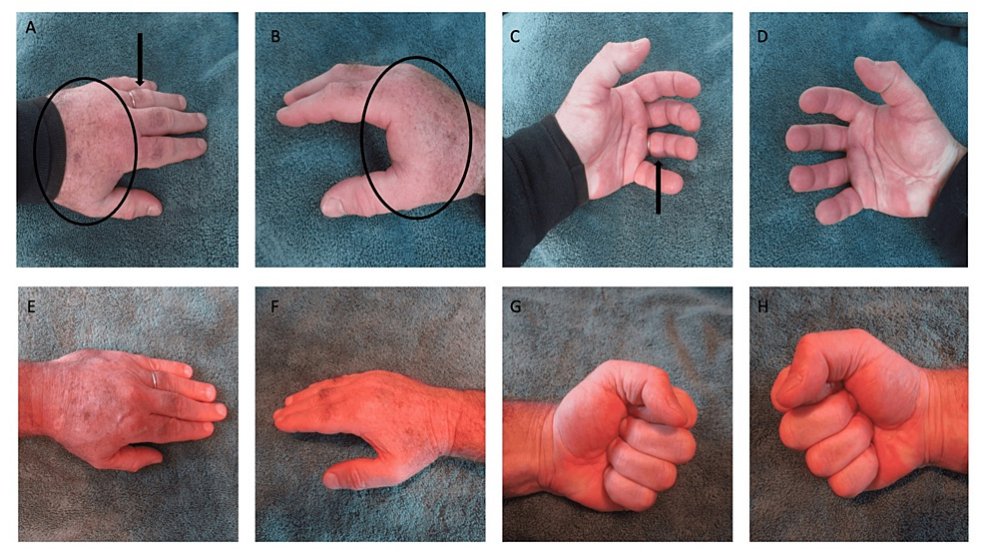
POTASH in a 65-year-old man (Case 1) Swelling of the hands and their digits began after one hour of walking. He completed the 13.1-mile half marathon after three hours and 53 minutes of walking; at that time, there was not only swelling of the left (A) and right (B) dorsal hands (surrounded by the black ovals) but also the digits. The dorsal view (A) and ventral view (C) of the left fourth finger show swelling of the digit distal to the ring (black arrows). Both the left (C) and right (D) hands had a positive fist sign; swelling of the palm and digits prevented him from making a fist. The ventral hand and digits became erythematous when he unsuccessfully attempted to clench his fingers into the palm of the hand (C and D). His POTASH completely resolved two hours later; the swelling is gone from the hands and fingers (E and F). In addition, after the swelling resolved, he now had a negative fist sign on both the left (G) and right (H) hand; he was able to tightly clench his fingers into the palm of each hand. POTASH: post ambulatory swollen hands

Clinical examination showed bilateral swollen hands and digits; the veins and tendons on his dorsal hands were not visible (Figure 1). This is especially evident on his left fourth finger distal to the ring that he is wearing. In addition, when he attempted to make a fist, he could not clench his fingers into the palm, which demonstrates a positive fist sign.

After he finished the race and stopped walking, the swelling stopped progressing. Within two hours after he was no longer walking, the swelling had spontaneously decreased. There was no swelling of his digits, particularly the left fourth finger distal to his ring. In addition, he had a negative fist sign and was now able to tightly bring the tips of his fingers into his palm and make a fist (Figure 1).

Case 2

A 58-year-old woman, the youngest sister of the man in Case 1, presented with asymptomatic swelling of her hands that developed while she was walking a half marathon in January 2023. She had initially experienced swelling of her hands 11 years earlier, at 47 years, when she would weekly walk up to 13 miles on the beach; the swelling begins after about one hour after she begins walking. There was no pruritus and no other sites of swelling.

**Figure 2:**
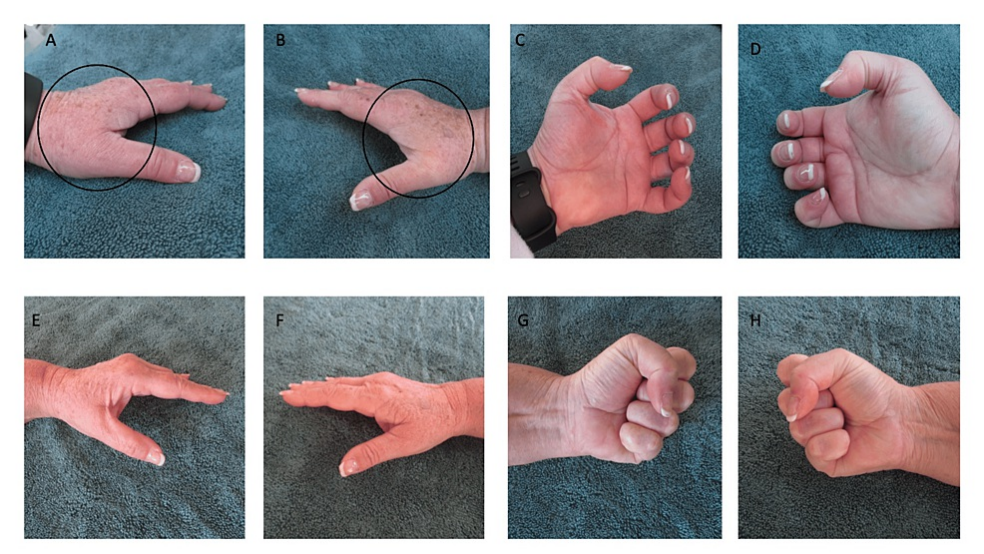
A 58-year-old woman (Case 2) with POTASH The youngest sister of the 65-year-old man also developed POTASH during the half marathon. The asymptomatic hand swelling began after an hour of walking; after completing the 13.1 miles in three hours and 43 minutes, her left (A) and right (B) hands had both developed prominent swelling (surrounded by the black ovals). She also had a positive fist sign (C and D) on her left (C) and right (D) hands. The swelling had completely resolved in both hands and their digits after two days (E and F). In addition, 48 hours after finishing the half marathon, she had a negative fist sign and could tightly clench her fingers into the palms of her left (G) and right (H) hands. POTASH: post ambulatory swollen hands

Cutaneous examination showed swelling of both palms and her fingers (Figure 2). She had a positive fist sign. She could not make a tight fist because of the edema in her digits.

The swelling spontaneously resolved after 48 hours (Figure 2). Thereafter, she was able to tightly clench her fingers into a fist. She now had a negative fist sign.

Case 3

The spouse of the man in Case 1 first developed asymptomatic swelling of her hands and fingers while running half marathons when she was in her 40s. Since then, she has had recurrent POTASH that begins about one hour after she starts running or walking. The patient and her husband have three adult biological children who have run half marathons, and none of them have POTASH.

**Figure 3:**
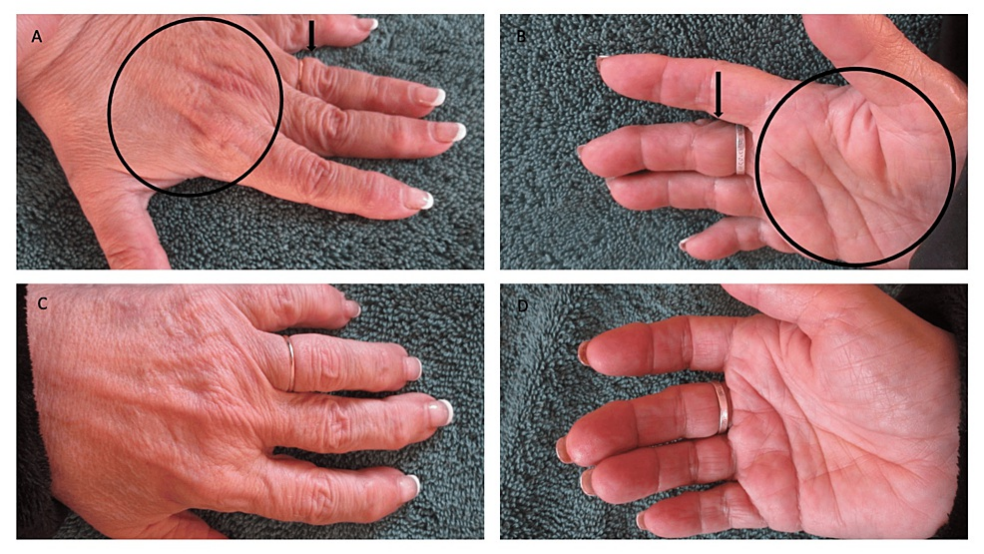
POTASH occurring in a woman (Case 3) The wife of the 65-year-old man also developed POTASH during the half marathon; she began to experience swelling of her hands after one hour of walking. After she completed the 13.1 miles, her dorsal left hand was swollen (A); both the tendons and superficial veins were less prominent (area surrounded by the black oval). In addition, her fingers were swollen (A); the digit on the fourth finger was swollen distal to the ring (black arrow). Her right palm (area surrounded by the black oval) also demonstrated swelling (B); not only the third digit was swollen distal to the ring on that finger (black arrow), but also there was swelling of the finger pads. Two hours after she stopped walking, the POTASH had resolved (C and D); the left (C) and right (D) hands and their digits were no longer swollen. POTASH: post ambulatory swollen hands

Cutaneous examination demonstrated swelling of the dorsum and the palms of both hands after completing a half marathon (Figure 3). The fingers and finger pads were also swollen. This is prominently demonstrated on the left fourth finger and the right third finger distal to the rings.

After she completed the race and stopped ambulating, the swelling began to spontaneously improve (Figure 3). Within two hours, the swelling of the hands and fingers had completely resolved.

## Discussion

The incidence of POTASH is more prevalent than the number of published papers in the medical literature [1,2,4,5]. The condition was initially described in 2011 [2]. Subsequently, it has only been reported by one of the authors in additional papers [1,4,5].

The condition initially appears in adulthood. Some of the individuals in this report first experienced swollen hands several years after when they began doing runs or walks of longer distances (Table 1). After the primary episode of POTASH, the hand swelling continued to subsequently appear each time the person ambulated for about an hour.

**Table 1:** Summary of clinical features of patients in this report of POTASH POTASH: post ambulatory swollen hands

Case	POTASH onset age	Gender	Duration of ambulation until POTASH	Itch	Other swelling sites	Time for spontaneous resolution
1	58 years	Male	1 hour	No	None	2 hours
2	47 years	Female	1 hour	No	None	48 hours
3	49 years	Female	1 hour	No	None	2 hours

Neither of the parents of the man and his sister had POTASH; similarly, their two other sisters, who both ran marathons, did not have POTASH. The man and his wife are a family, yet not blood-related. However, none of their three adult children who have run several half marathons have developed POTASH.

The etiology of POTASH is undefined [1,2,4]. Whether the condition is merely acquired or inherited or both has not yet been elucidated. The pattern of occurrence in the man's birth family and current family raises the possibility of an autosomal recessive pattern of inheritance, although this could be a sporadic occurrence.

## Conclusions

Walking, hiking, or running may be associated with swelling of the hands and their digits; this acquired condition is referred to as post ambulatory swollen hands or its acronym POTASH. The recurrent ambulation-related asymptomatic swelling only affects the hands, thumbs, and fingers. Frequently, the affected individual is not able to make a fist; this is designated as a positive fist sign. Within a few hours after stopping ambulation, POTASH typically resolves spontaneously; yet, in some individuals, it can persist for one or two days. The etiology of POTASH remains to be established. Three individuals with POTASH are reported: a man, one of his younger sisters, and his wife. Neither the man's parents nor two of his other sisters had POTASH. In addition, the three biological adult children of the man and his wife did not have POTASH. In conclusion, it is possible that POTASH may be a sporadic condition; alternatively, a genetic predisposition, such as an autosomal recessive mode of inheritance, may contribute to the pathogenesis of POTASH in some of the patients with this condition.
